# Exclusive Breastfeeding or Formula Use? A Cross-Sectional Survey of Romanian Mothers’ Feeding Practices and Influencing Factors

**DOI:** 10.3390/medicina61081425

**Published:** 2025-08-07

**Authors:** Ioana Roșca, Andreea Teodora Constantin, Alexandru Dinulescu, Mirela-Luminița Pavelescu, Leonard Năstase, Daniela-Eugenia Popescu, Alexandru Blidaru

**Affiliations:** 1 Faculty of Midwifery and Nursery, University of Medicine and Pharmacy “Carol Davila”, 020021 Bucharest, Romania; ioana.rosca@umfcd.ro; 2 Neonatology Department, Clinical Hospital of Obstetrics and Gynecology “Prof. Dr. P.Sârbu”, 060251 Bucharest, Romania; 3 Pediatrics Department, National Institute for Mother and Child Health “Alessandrescu-Rusescu”, 020395 Bucharest, Romania; 4 Faculty of Medicine, University of Medicine and Pharmacy “Carol Davila”, 050474 Bucharest, Romaniaalexandru.blidaru@umfcd.ro (A.B.); 5 Emergency Hospital for Children “Grigore Alexandrescu”, 011743 Bucharest, Romania; 6 Neonatology Department, National Institute for Mother and Child Health “Alessandrescu-Rusescu”, 011061 Bucharest, Romania; 7 Department of Obstetrics-Gynecology and Neonatology, “Victor Babeș” University of Medicine and Pharmacy, 300041 Timisoara, Romania; 8 Department of Neonatology, Premiere Hospital, Regina Maria Health Network, 300645 Timisoara, Romania; 9 Institute of Oncology “Prof. Dr. Alexandru Trestioreanu”, 022314 Bucharest, Romania

**Keywords:** breastfeeding, formula, motherhood, neonatology, pediatrics

## Abstract

*Background and Objectives:* Exclusive breastfeeding offers optimal nutrition and health benefits for infants, yet many mothers face challenges that impact their ability to breastfeed. This study aimed to explore breastfeeding practices among Romanian mothers and identify factors associated with successful exclusive breastfeeding. *Materials and Methods:* A cross-sectional online survey was conducted from February to March 2025, targeting Romanian mothers via social media platforms. The questionnaire, developed specifically for this study, collected data on sociodemographics, birth and neonatal variables, hospital practices, feeding intentions, community influences, and breastfeeding outcomes. A total of 874 valid responses were analyzed using Fisher’s exact tests and multivariable logistic regression. *Results:* While 87.2% of mothers intended to breastfeed, only 56.1% reported exclusive breastfeeding. Factors significantly associated with reduced likelihood of exclusive breastfeeding included maternal age ≥ 30 years (OR = 1.40, *p* = 0.042), Cesarean delivery (OR = 1.78, *p* < 0.001), absence of rooming-in (OR = 2.32, *p* < 0.001), and pacifier use (OR > 4.7, *p* < 0.001). Protective factors included non-smoking status (OR = 0.52, *p* < 0.001) and encouragement to breastfeed by medical staff (OR = 1.60, *p* = 0.004). Despite external advice to use formula, many mothers continued breastfeeding. *Conclusions:* Although breastfeeding intention was high, exclusive breastfeeding remains suboptimal in Romania. Targeted support—particularly in maternity hospitals and for mothers recovering from Cesarean sections—alongside prenatal education and consistent postnatal guidance are essential to bridge the gap between intention and practice.

## 1. Introduction

Breastfeeding is widely recognized by leading global health organizations as the optimal source of nutrition and protection for infants, offering a myriad of unparalleled benefits for both baby and mother. The World Health Organization (WHO) unequivocally recommends exclusive breastfeeding for the first six months of life, followed by continued breastfeeding alongside appropriate complementary foods for up to two years or more. This recommendation is echoed by the American Academy of Pediatrics (AAP), which emphasizes the critical role of human milk in promoting infant health and development, reducing the risk of numerous childhood illnesses, and fostering maternal well-being [1,2,3]. These recommendations are supported strongly by the other multiple medical and professional organizations, such as the American Academy of Family Physicians (AAFP), American College of Obstetricians and Gynecologists (ACOG), and Canadian Pediatric Society (CPS), based on both short- and long-term benefits for the mother and child [4,5].

Globally, exclusive breastfeeding (EBF) rates reached 48% in 2023 for infants under six months while the 2030 Global Nutrition Target is set at 70% [6,7]. Nationally representative data on exclusive breastfeeding in Romania remain limited. One study indicated that only 12.6% of infants were exclusively breastfed at 6 months in 2011 with a modest rise to 29.8% in 2016 while a 2021 study in northwest Romania reported a 46.7% exclusive breastfeeding prevalence [8,9].

Previous studies in various populations have identified multiple factors associated with exclusive breastfeeding, including maternal age, education level, smoking status, delivery mode, prenatal education, family support, and hospital practices such as rooming-in and immediate skin-to-skin contact [9,10,11,12].

As noted in that 2021 study [9], there are few up-to-date data available on breastfeeding practices in Romania. To this day, in our country, evidence on the specific influences shaping maternal feeding practices remains scarce. Recent policy interest in improving maternal–child outcomes—alongside changing birth practices and marketing of formula—necessitates an updated evaluation of how Romanian mothers experience and navigate early infant feeding decisions.

Beyond its nutritional superiority, breastfeeding is a powerful public health tool, contributing to reduced infant mortality, improved cognitive development, and a lower incidence of chronic diseases later in life. As such, promoting and supporting breastfeeding is not merely a personal choice, but a collective responsibility with profound implications for the health and prosperity of future generations.

This study aimed to assess the prevalence of exclusive breastfeeding and to identify sociodemographic, clinical, and contextual factors associated with feeding choices among Romanian mothers. Our findings aim to inform clinical practices and public health interventions tailored to the local context.

## 2. Materials and Methods

### 2.1. Study Setting

We conducted a cross-sectional observational study in Romania, based on responses to an online survey regarding breastfeeding practices. Participants were recruited between February and March 2025 through non-probability convenience sampling, by targeting online parenting communities, both from urban and rural areas. Platforms used included Facebook, Instagram, and WhatsApp groups.

### 2.2. Study Population and Eligibility Criteria

Eligible participants were mothers of at least one child who expressed willingness to complete the online questionnaire. The questionnaire was not addressed to pregnant women without prior children. However, a small number of respondents who were pregnant with their second child and already had breastfeeding experience were included. Responses were excluded from the analysis if the submission was incomplete.

### 2.3. Study Sample

A total of 874 complete and valid responses were obtained during the one-month recruitment period. The required sample size was estimated using a standard formula for proportions, assuming a 95% confidence level, a 5% margin of error, and an expected proportion of exclusive breastfeeding of 56.1%, based on prior national reports. The minimum required sample size was calculated to be 380 participants. With 874 complete and eligible responses, the actual study sample exceeded this threshold, providing adequate statistical power for both descriptive and inferential analyses, including subgroup comparisons and multivariable logistic regression.

The final sample consisted of mothers who responded completely and met eligibility criteria. Participants were instructed to respond with reference to their youngest child. No identifying data were collected, in accordance with GDPR regulations, ensuring anonymity.

### 2.4. Questionnaire Development and Validation

A self-administered online questionnaire was developed specifically for this study, inspired by existing surveys on breastfeeding practices and attitudes [13,14,15,16,17], adapted to reflect the Romanian cultural and healthcare context. Face validity was evaluated through pre-testing with five mothers with recent breastfeeding experience. Their feedback on clarity, content, and length was used to refine the final version of the survey.

Although the questionnaire was informed by previous instruments, no formal psychometric validation (such as construct validity or reliability testing) was conducted, as the study was exploratory in nature and aimed to collect descriptive data within a limited timeframe.

Both single- and multiple-response questions were used depending on the structure of the variable: mutually exclusive categories (e.g., type of delivery) were addressed with single-choice questions, while complex behaviors and influences (e.g., reasons for supplementing with formula, sources of advice) were assessed using multiple-response formats. No separate qualitative study was conducted beforehand; however, answer options were informed by findings from the literature and by the clinical and community experiences of the research team.

The survey was available in Romanian and required approximately 5 min to complete.

### 2.5. Variables Collected

The questionnaire covered the following domains:

- Sociodemographic data: maternal age, education level, family income, area of residence (urban/rural), smoking status, and number of children.
- Perinatal factors: maternal age at first birth, birth method (vaginal or Cesarean), gestational age, birth weight, and timing of first skin-to-skin contact.
- Hospital feeding practices and support: rooming-in status, type of feeding in hospital, advice and support received from medical staff regarding breastfeeding initiation and technique, and use of pacifiers.
- Breastfeeding behavior and intentions: whether the mother planned to breastfeed, initiation and duration of breastfeeding, timing of milk production onset, and whether difficulties were encountered (e.g., latch problems, pain, mastitis, perceived low supply).
- Use of formula: whether the infant received formula and at what point, and whether there was any external pressure (from family, medical staff, or community) to introduce formula.
- Attitudes and perceptions: maternal experience with breastfeeding (rated from very positive to very difficult), perceived facilitators of successful breastfeeding (e.g., partner support, lactation consultants), and sources of information (e.g., prenatal classes, online forums, medical professionals).

### 2.6. Data Collection

Data were collected via an anonymous online questionnaire distributed between February and March 2025. Participation was voluntary, no identifiable data were gathered.

The International Standard Classification of Education (ISCED) adapted to our country (Romania) was used to classify the education of the mothers [18]. See Table 1 for details.

Family income was classified as low, middle, and high by the latest reports of the Romanian National Institute of Statistics [19,20]. A detailed comparison is provided in Table 2.

### 2.7. Statistical Analysis

Data were analyzed using IBM SPSS Statistics version 26. Fisher’s exact test was used independently to assess the association between the type of feeding (breastfeeding, mixt feeding and exclusive formula) and several variables: mother age group, education and income level, maternal smoking status, attendance at prenatal breastfeeding courses, type of delivery, age of the child at birth, breastfeeding encouragement by the medical staff when first holding the baby and rooming-in.

A binary multivariate logistic regression was performed that compared “breastfeeding” and “mixed/exclusive formula”. The predictors used were as follows:

- Age the mother had her child (<30 years vs. ≥30 years).
- Education level (low/medium vs. high), income level (low/middle vs. high), maternal smoking status (yes/no).
- Attendance at prenatal breastfeeding courses (yes/no).
- Type of delivery (vaginal vs. C-section).
- Age at birth (preterm vs. term).
- Breastfeeding encouragement by the medical staff when first holding the baby (yes/no).
- Community pressure, the use of pacifier (no vs. yes from birth vs. yes after 6 weeks) and rooming-in (yes/no).

The selection of variables was based both on their statistical significance in a univariate analysis and on prior evidence from the literature regarding their relevance to breastfeeding outcomes. Additionally, variables such as pacifier use and community pressure were included based on empirical observations from our clinical practice, reflecting common concerns encountered among Romanian mothers. The model fits were evaluated using the Hosmer and Lemeshow test, which indicated that the model sufficiently fit the data (*p* = 0.707).

### 2.8. Ethics

The study was approved by the Ethics Committee of the Clinical Hospital of Obstetrics and Gynecology “Prof. Dr. Panait Sîrbu”, Bucharest, Romania, under approval number 5828/08.04.2025 and was conducted respecting the Helsinki Declaration for Human Rights.

## 3. Results

A total of 874 responses were collected during the one-month data collection period (February–March 2025). Exclusive breastfeeding was defined for participants as feeding the infant only breast milk, with no additional water, tea, formula, or other liquids or solids, in accordance with WHO guidelines.

### 3.1. Participant Characteristics

The majority of respondents were between 30 and 40 years old (58.7%), urban residents (75.7%), and had a high education level (76.2%). Most were non-smokers (72.1%). These sociodemographic characteristics are detailed in Table 3.

### 3.2. Obstetric and Neonatal Background

Only 32.2% of respondents gave birth vaginally, while the majority (60.3%) delivered via Cesarean section without general anesthesia, and 7.6% via Cesarean section with general anesthesia. Among those who had a Cesarean, the most common reason was a planned procedure (54.1%), followed by medical emergencies (28.5%), and previous Cesarean delivery (12.0%). Most babies (87%) were born at term (>37 weeks gestation), and 85.8% had a birth weight between 2500 g and 4000 g.

### 3.3. Initial Breastfeeding Practices

Only 27.1% of mothers held their baby immediately after birth, and 64.3% reported being encouraged by maternity staff to initiate breastfeeding. Regarding the timing of first breastfeeding, 22.5% of mothers put the baby to the breast more than 24 h after birth, while only 7.9% did so immediately.

During the hospital stay, 66% of newborns stayed with their mothers in a rooming-in arrangement. Breastfeeding support was offered to 73% of participants, although 25.3% did not receive any such assistance. During their hospital stay at birth, most infants were mixed-fed (57.8%), followed by direct breastfeeding (19.7%) and feeding with expressed breast milk via syringe or bottle (18.8%).

### 3.4. Breastfeeding Intention and Support

Before delivery, 87.2% of mothers planned to breastfeed, while 4.8% did not, and 8% were undecided. Despite this high intention rate, only 29.6% of respondents accessed support from a certified lactation consultant, with cost being a limiting factor for 13.6% of those who would have wanted assistance.

Support during hospitalization varied: although 73% received some help with breastfeeding, and 64.3% recalled being actively encouraged to initiate breastfeeding by the medical staff, Notably, 49% of mothers reported being told by someone in their community that they might not have “good milk” or were “not good at breastfeeding”, and 54.7% were advised by family or acquaintances to give formula, based on beliefs that it would improve sleep or weight gain. In contrast, only 10% reported pressure from medical professionals to cease breastfeeding.

### 3.5. Feeding Methods and Duration

Regarding actual feeding practices, 56.1% of mothers reported exclusive breastfeeding, 22.7% practiced mixed feeding (breast milk and formula), and 21.3% used formula exclusively. When asked about duration, 35.2% breastfed for 13–24 months, and 23.8% continued beyond 24 months. Only 2.5% breastfed for less than one month.

Among those who encountered breastfeeding difficulties (72.1% of all respondents), the most commonly reported issues included nipple pain (56.8%), improper latch (56.4%), perceived insufficient milk (31.5%), and severe breast engorgement (40.8%). Mastitis or other infections were reported by 13.4%, while 11.9% cited a lack of family support.

### 3.6. Contributors to Breastfeeding Success

Respondents identified several factors contributing to successful breastfeeding. The most frequently mentioned were partner/family support (52.5%), information received in the maternity hospital (21.1%), and previous experience with breastfeeding (28.8%). Personal determination and access to mother support groups were also noted as helpful.

### 3.7. Infant-Related Challenges

Only 10.8% of respondents reported that their baby had a condition that negatively impacted breastfeeding. Among these, the most common were tongue-tie (frenulum issues), prematurity, and gastroesophageal reflux. Other mentioned problems included weak suck, neonatal jaundice, and structural or neurological issues (e.g., high palate, hypotonia, Down syndrome).

### 3.8. Factors Associated with the Type of Feeding

Table 4 summarizes the associations between selected sociodemographic, behavioral, perinatal, and hospital-related variables and the type of infant feeding (exclusive breastfeeding vs. mixed/formula feeding). Statistically significant associations were identified for maternal age, education level, income, smoking status, participation in prenatal breastfeeding courses, type of delivery, gestational age, breastfeeding encouragement by medical staff, rooming-in, community pressure, and pacifier use (all *p* < 0.05).

Exclusive breastfeeding was significantly more frequent among non-smoking mothers (60.8%), those who attended prenatal courses (62.4%), delivered vaginally (66.5%), practiced rooming-in (63.3%), and avoided early pacifier use (77.2% among those who never used pacifiers). Conversely, mixed or formula feeding was more prevalent among mothers aged over 40, those who delivered via Cesarean section, and those who introduced pacifiers from birth or before six weeks. Encouragement by medical staff and support from the community were also associated with higher exclusive breastfeeding rates.

Significant associations were observed between the type of feeding and the following variables: age the mother had her child, education level, income level, maternal smoking status, attendance at prenatal breastfeeding courses, type of delivery, age of birth, breastfeeding encouragement by the medical staff when first holding the baby, community pressure, and rooming-in, variables that were used further in the regression (summary in Table 5).

The significant predictors (*p* < 0.05) were as follows:

- Age of the mother (≥30 years) (OR = 1.40; 95% CI: 1.01–1.93; *p* = 0.042). Mothers aged ≥30 years were 1.4 times more likely to use mixed/formula feeding compared to younger mothers.
- Maternal smoking status (non-smoker) (OR = 0.52; 95% CI: 0.37–0.73; *p* < 0.001). Non-smokers were less likely to use mixed/formula feeding (protective factor for breastfeeding).
- Type of delivery (C-section) (OR = 1.78; 95% CI: 1.27–2.49; *p* = 0.001). Mothers who had a C-section were 1.78 times more likely to use mixed/formula feeding.
- Breastfeeding encouragement by medical staff (yes) (OR = 1.60; 95% CI: 1.16–2.20; *p* = 0.004). While medical encouragement is typically a protective factor, in this case it may reflect intervention in response to early difficulties, suggesting reverse causality.
- Rooming-in (not practiced) (OR = 2.32; 95% CI: 1.67–3.22; *p* < 0.001). Infants not roomed-in had over 2.32 times the odds of receiving mixed/formula feeding.
- Advised by community to give formula (OR = 0.673; 95% CI [0.461–982]; *p* = 0.04). Mothers who were advised to give formula had 33% lower odds of actually using it.
- Offering pacifier from birth and after 6 weeks both significantly reduced the likelihood of exclusive breastfeeding (OR = 5.03 and 4.77, respectively; *p* < 0.001).

## 4. Discussion

In this cross-sectional survey of 874 Romanian mothers, exclusive breastfeeding was reported by 56.1%. A multivariate analysis identified advanced maternal age (≥30 years), Cesarean delivery, absence of rooming-in, and pacifier use as independent risk factors for mixed/formula feeding, while smoking was a significant negative predictor.

According to the World Health Organization, rates of exclusive breastfeeding in the first 6 months of life have increased globally and reached 48% in 2023 [6]. In Romania, exclusive breastfeeding seems to follow a similar rising trend. A study conducted in 2021 on breastfeeding and diversification attitudes among Romanian mothers reported a prevalence of 32.18% for breastfeeding for more than 6 months [21].

A contributing factor for the relatively low exclusively breastfeeding rate may be the higher rate of newborns delivered via C-section (67.9%), which often prevents immediate skin-to-skin contact for newborns. When asked, ‘When did you first hold your baby?’, a significant percentage of mothers responded, ‘after several hours’, which may account for the delayed initiation of breastfeeding. Early skin-to-skin contact between mothers and newborns has some short-term neurobehavioral benefits and may program other benefits during this sensitive period of adaptation to extrauterine life. In the short term, early skin-to-skin contact appears to reduce infant crying, increase blood glucose levels, and promote greater cardiorespiratory stability in late preterm infants [22]. Early skin-to-skin contact also helps to establish lactation and promote ongoing breastfeeding, which enhances the other benefits of breastfeeding outlined below [3,22].

According to the study, mothers who delivered vaginally were less likely to use formula-only or mixed feeding compared to those who delivered via Cesarean section. The rate of exclusive breastfeeding was highest in the vaginal birth group, although not statistically different across groups after correction. These findings support the idea that Cesarean sections—especially without general anesthesia—may pose barriers to optimal breastfeeding).

The observed lack of immediate postpartum contact and inconsistent breastfeeding support highlight the need to align Romanian maternity care with the Baby-Friendly Hospital Initiative (BFHI). This WHO/UNICEF program promotes evidence-based practices such as early skin-to-skin, rooming-in, and trained lactation support, all of which were shown to improve breastfeeding outcomes in other countries [23].

A large proportion of mothers reported encountering difficulties with breastfeeding when surveyed, indicating a need for support to sustain breastfeeding. Maternal–neonate separation constitutes a source of toxic stress with profound long-term negative implications for health and development; it is imperative to rethink standard neonatal care practices. Adopting a “zero separation” paradigm, prioritizing immediate and continuous skin-to-skin contact from birth, becomes essential to support optimal child development and prevent later health problems, including those explained by the Developmental Origins of Health and Disease [24].

Our study findings indicate that younger mothers tend to rely more on formula milk, while older mothers, especially those in the 30–40 age group, prefer exclusive breastfeeding. Intention to breastfeed is a strong predictor of initiation and duration of breastfeeding [11,25,26,27]. Despite the high intention to breastfeed observed in our sample (87.2%), only 56.1% reported exclusive breastfeeding. This highlights a well-known “intention–behavior gap”, frequently cited in breastfeeding literature. Mothers may face unanticipated barriers after birth—such as pain, fatigue, lack of support, or misinformation—that override their initial intention, especially during the first 48–72 h postpartum [28]. New parents are more likely to intend to breastfeed if they have a previous successful breastfeeding experience and if this decision is supported by family, community, and workplace. Conversely, a parent is less likely to intend to breastfeed if she is facing economic challenges (lower household income and/or needing to return to work), is pregnant with twins, is younger, and/or has limited access to health care [10,12,27].

The association between the education level and the type of feeding was statistically significant. These findings suggest that a higher level of maternal education is associated with an increased likelihood of exclusive breastfeeding. Breastfeeding offers short- and long-term health and developmental benefits to children and their mothers. Women require support from all of society to fulfill their breastfeeding plans [29,30]. However, their level of understanding and awareness is commensurate with their level of education.

Our results suggest that family income may play a role in shaping infant feeding decisions, with lower-income mothers more frequently relying on formula compared to those from middle-income households, which is absurd, as it is precisely those with lower incomes who should benefit from free breast milk. This shows that there is a strong link between education level and family income. Understanding which factors affect parental choices about infant feeding is essential to providing appropriate education and support. In addition, counseling is enhanced by recognizing common misconceptions and barriers about breastfeeding and how to overcome them. Similarly, public resources and policy should be directed at removing and addressing the common obstacles to breastfeeding in a population [12,27,30].

We examined the association between maternal smoking status and infant feeding practices. These findings suggest that maternal smoking is associated with a lower likelihood of exclusive breastfeeding and a higher reliance on formula feeding, underscoring the potential impact of tobacco use on infant nutrition choices. Breastfeeding in high-resource countries is associated with higher socioeconomic and educational status and lower rates of maternal obesity and smoking [3,31]. Other factors that decrease milk volume include maternal smoking, stress, anxiety, fatigue, and illness [27,32,33]. Marketing of artificial breast milk substitutes (formulas) often has a negative influence on breastfeeding initiation [27,34].

Attending prenatal breastfeeding courses appears to have a positive impact on infant feeding practices. Specifically, mothers who participated in such education were significantly less likely to feed their babies exclusively with formula and more likely to exclusively breastfeed, suggesting that these programs may play an important role in encouraging breastfeeding, especially exclusive breastfeeding. Expectant parents often make decisions about how they will feed their infant very early in pregnancy or before conceiving [27,35]. Routine ongoing support and guidance during antenatal and postnatal care are associated with longer duration of breastfeeding (exclusive and partial). This support is optimally tailored to the setting and needs of the population and individual patient and may include a variety of professional or lay/peer counselors. All birthing parents should have an initial assessment and counseling regarding breastfeeding by a clinician with experience in this area. This could be an obstetrician or midwife, general practitioner or family medicine clinician, pediatrician, or lactation consultant [26,27,35].

The central role of partner involvement in breastfeeding success suggests that educational efforts should not focus solely on mothers. Fathers and other family members influence both practical and emotional aspects of breastfeeding, especially in moments of uncertainty or fatigue. Integrating them into antenatal counseling could help normalize shared responsibility and counteract negative social messaging [36].

Prematurity was the most frequent reason mothers in the study group could not breastfeed. This is consistent with existing evidence showing that mothers of preterm infants face unique challenges. Beyond logistical difficulties, prematurity is often accompanied by maternal psychological stress, which may physiologically interfere with lactation. Elevated levels of stress hormones such as cortisol and adrenaline can suppress oxytocin release, inhibit the milk ejection reflex, and delay lactogenesis [37,38,39]. Moreover, the frequent separation of mother and infant in neonatal intensive care units limits opportunities for skin-to-skin contact and direct suckling, further impacting milk supply and breastfeeding success. However, in maternity hospitals, we can encourage administering expressed breast milk in situations where the newborn is in intensive care. Many mothers are unable to provide sufficient mother’s milk to meet the needs of their preterm infant during the birth hospitalization and following discharge. In the United States, approximately one-half of very-low-birth-weight (VLBW) infants were being fed human milk (primarily mother’s own milk but sometimes donor human milk) at the time of hospital discharge [40,41]. Premature infants have greater nutritional needs in the neonatal period than at any other time of their lives. The nutrient needs are inherently high at this stage of development to match the high rates of nutrient deposition achieved by infants in utero [40,42].

To significantly boost breastfeeding rates in Romania, particularly for vulnerable newborns, focused support programs for mothers of premature infants are crucial. These programs should go beyond general breastfeeding education to include practical assistance with expressed breast milk administration while their babies are in neonatal intensive care units (NICUs). This can involve providing access to high-quality breast pumps, dedicated lactation consultants within NICUs, and clear guidance on safe milk collection, storage, and delivery. By empowering these mothers with the knowledge and tools to provide breast milk, even when direct breastfeeding is not immediately possible, we can significantly improve the health outcomes for premature babies and establish a foundation for continued breastfeeding post-discharge. Pacifier use was one of the strongest predictors of non-exclusive breastfeeding in our sample. Early introduction (from birth) and even delayed use (after six weeks) were both significantly associated with increased odds of mixed or formula feeding. Several studies have raised concerns that pacifiers may interfere with breastfeeding by altering sucking patterns, reducing feeding frequency, or leading to nipple confusion, particularly in the early postpartum period [43,44]. The WHO and UNICEF discourage routine pacifier use during the establishment of breastfeeding, especially in hospital settings, as part of the Baby-Friendly Hospital Initiative [23]. However, other studies, including large observational cohorts and Cochrane reviews, have found that pacifier use does not necessarily reduce breastfeeding duration when breastfeeding is already established and may have benefits such as reduced risk of sudden infant death syndrome (SIDS) [45,46,47]. Therefore, recommendations regarding pacifier use should consider timing, maternal intent, and context. In light of our findings, caution should be exercised with pacifier introduction, especially in the first weeks postpartum when lactation is most vulnerable.

To significantly elevate breastfeeding rates and improve public health outcomes in Romania, a comprehensive and integrated national strategy encompassing robust education and health policies is essential. This strategy must move beyond fragmented initiatives to establish a cohesive framework that begins with early, consistent, and evidence-based education for expectant parents, healthcare professionals, and the wider community. This education should not only highlight the unparalleled health benefits of breastfeeding for both mother and child but also address common misconceptions and provide practical support. Concurrently, national health policies must be strengthened to mandate and support breastfeeding-friendly practices within all healthcare institutions, including the implementation of the Baby-Friendly Hospital Initiative. This includes adequate training for medical staff, ensuring access to qualified lactation consultants, and establishing clear guidelines for the ethical marketing and use of breast milk substitutes. By embedding breastfeeding support into the fabric of both our educational system and healthcare infrastructure, Romania can foster a culture where breastfeeding is not just encouraged but actively facilitated and celebrated as a cornerstone of maternal and child well-being.

The results of our study highlight the crucial importance of professional support and promotion of breastfeeding. It highlights the significant role of several categories of professionals and national programs:

- The need for comprehensive postpartum follow-up programs that should be available in all health care systems and include the following: nurses, pediatricians, obstetricians, lactation consultants, and, sometimes, psychologists. This promising package would be beneficial in helping new families get off to a good start with breastfeeding.
- Specialized professionals present in maternity hospitals: doctors specializing in breastfeeding medicine, internationally certified lactation consultants, and breastfeeding counselors to provide expertise, education and individualized support for each mother.
- Creating a national program of home visits by midwives and community health workers helping postpartum mothers overcome initial breastfeeding difficulties.
- Implementation of “Baby Friendly Hospital” and “Breastfeeding Friendly Work Environment” programs in all maternity hospitals and neonatology wards.
- Improving prenatal education on breastfeeding: prenatal courses, offered free of charge or reimbursed by the state, organized in maternity hospitals.
- Telephone support: Telephone helplines can support breastfeeding and increase individual efficacy by encouraging mothers to seek help for breastfeeding issues.

This study has several limitations. First, its cross-sectional design precludes causal inference, limiting interpretation to associations rather than directional effects. Second, the self-administered online survey may be subject to recall and social desirability bias, particularly regarding sensitive behaviors such as smoking or feeding practices. Third, the sample is skewed toward urban, educated, and middle-income mothers, potentially limiting generalizability to more vulnerable or underserved populations. Although the questionnaire underwent face validation through pilot testing with five mothers, no formal psychometric validation (e.g., construct validity, reliability testing) was conducted. This limits the ability to generalize measurement precision. Finally, the low representation of mothers with minimal education (*n* = 11) reduces the statistical power to detect differences in this subgroup.

## 5. Conclusions

Our study highlights that exclusive breastfeeding in Romania remains suboptimal and is significantly influenced by factors such as medical staff encouragement, rooming-in practices, pacifier use, maternal education, and societal attitudes. These findings emphasize the crucial role of professional and community support in shaping maternal feeding decisions. Strengthening breastfeeding-friendly policies, ensuring access to specialized guidance, and promoting early prenatal education can help improve breastfeeding rates. A coordinated national effort is needed to support mothers throughout the perinatal period and promote breastfeeding as a public health priority.

## Figures and Tables

**Table 1:** International Standard Classification of Education (ISCED) adapted to the Romanian educational system.

Education Level	ISCED	Score
Low education	Early childhood education (‘less than primary’ for educational attainment)	0
	Primary education	1
	Lower secondary education	2
Medium education	Upper secondary education	3
	Post-secondary non-tertiary education	4
High education	Short-cycle tertiary education	5
	Bachelor’s or equivalent level	6
	Master’s or equivalent level	7
	Doctoral or equivalent level	8

**Table 2:** Family income categories.

Family Income Category	Monthly Net Income (RON *)	Monthly Net Income (€ *)	Typical Characteristics
Low income	<6000	<~1208	Single-income households, minimum wage or low-skilled jobs, basic needs may be unmet
Middle income	6000–16,000	~1208–3220	Dual-income households, professionals or skilled workers, able to cover expenses and save modestly
High income	>16,000	>~3220	High-skilled professionals, entrepreneurs, or corporate roles; higher savings and consumption capacity

* RON = Romanian New Leu; € = Euro.

**Table 3:** Descriptive statistics of main population characteristics.

Variable	*n* (%)
Age (years)	
<18 years old	2 (0.2%)
18–20 years old	6 (0.7%)
20–30 years old	248 (28.4%)
30–40 years old	513 (58.7%)
Over 40 years old	105 (12%)
Area of provenance	
Urban	662 (75.7%)
Rural	212 (24.3%)
Mother education	
High education	666 (76.2%)
Medium education	197 (22.5%)
Low education	11 (1.3%)
Smoking status	
Smoker	244 (27.9%)
Non-smoker	630 (72.1%)
Delivery type	
Vaginally	281 (32.2%)
C-section without general anesthesia	527 (60.3%)
C-section with general anesthesia	66 (7.6%)
Holding the baby after birth	
Immediately	237 (27.1%)
5 min after birth	76 (8.7%)
30 min or more	34 (3.9%)
After 1 h	57 (6.5%)
After several hours	449 (51.4%)
Does not know when	21 (2.4%)
Timing of first breastfeeding	
Immediately	69 (7.9%)
One hour after birth	128 (14.6%)
Less than 12 h after birth	251 (28.7%)
More than 12 h after birth	205 (23.5%)
More than 24 h after birth	197 (22.5%)
Never breastfed	24 (2.7%)
Rooming-in during hospital stay	
Yes	577 (66%)
No	297 (34%)
Pacifier use	
Since birth	286 (32.7%)
After 6 weeks	220 (25.2%)
No	368 (42.1%)

**Table 4:** Factors associated with the type of feeding.

	Exclusive Breastfeeding	Mixed/Exclusive Formula Feeding	Association (Fisher’s Exact Test) (*p*)
	490 (56.1%) *	384 (43.9%)	
Age group			0.005
<18 years old	5 (41.7%)	7 (58.3%)	
18–20 years old	15 (48.4%)	16 (51.6%)	
20–30 years old	**303 (60%)**	**202 (40%)**	
30–40 years old	164 (52.6%)	148 (47.4%)	
Over 40 years old	**3 (21.4%)**	**11 (78.6%)**	
Education group			0.002
High education	**384 (57.7%)**	**282 (42.3%)**	
Medium education	**103 (52.3%)**	**94 (47.7%)**	
Low education	**3 (27.3%)**	**8 (72.7%)**	
Income			0.044
High income	**91 (53.8%)**	**78 (46.2%)**	
Middle income	**302 (58.2%)**	**217 (41.8%)**	
Low income	**97 (52.2%)**	**89 (47.8%)**	
Maternal smoking status			<0.001
Smoker	**107 (43.9%)**	**137 (56.1%)**	
Non-smoker	**383 (60.8%)**	**247 (39.2%)**	
Attendance at prenatal breastfeeding courses			=0.010
Yes	**116 (62.4%)**	**70 (37.6%)**	
No	**374 (54.4%)**	**314 (45.6%)**	
Type of delivery			<0.001
Cesarean section	**303 (51.1%)**	**290 (48.9%)**	
Vaginal birth	**187 (66.5%)**	**94 (33.5%)**	
Gestational age			0.001
>37 weeks	**435 (57.2%)**	**325 (42.8%)**	
34–37 weeks	**48 (56.5%)**	**37 (43.5%)**	
32–34 weeks	**1 (10.0%)**	**9 (90.0%)**	
28–32 weeks	**3 (25.0%)**	**9 (75%)**	
<28 weeks	**3 (42.9%)**	**4 (57.1%)**	0.015
Breastfeeding encouragement by the medical staff when first holding the baby			
Yes	**335 (59.6%)**	**227 (40.4%)**	
No	**155 (49.7%)**	**157 (50.3%)**	
Rooming-in			<0.001
Yes	**365 (63.3%)**	**212 (36.7%)**	
No	**125 (42.1%)**	**172 (57.9%)**	
Were you told by community members that your breast milk was not good?			0.004
Yes	**233 (60.1%)**	**155 (39.1%)**	
No	**257 (52.9%)**	**229 (47.1%)**	
Advised by the community members to give formula			<0.001
Yes	**225 (63.6%)**	**129 (36.4%)**	
No	**265 (51%)**	**255 (49%)**	
Use of pacifier			<0.001
No	**284 (77.2%)**	**84 (22.8%)**	
Yes, from birth	**115 (40.2%)**	**171 (59.8%)**	
Yes, after 6 weeks	**91 (41.4%)**	**129 (58.6)**	

* The bold values are the statistically significant groups after Bonferroni correction.

**Table 5:** Logistic regression for the type of feeding (breastfeeding vs. mixed/exclusive formula feeding).

Predictor	OR (95% CI)	*p*
Age of the mother at birth (≥30 years)	1.398 (1.012–1.931)	=0.042 *
Education level (high)	0.851 (0.586–1.234)	=0.394
Income level (high)	1.160 (0.783–1.719)	=0.459
Maternal smoking status (non-smoker)	0.516 (0.367–0.726)	**<0.001**
Attendance at prenatal breastfeeding courses (yes)	0.761 (0.517–1.118)	=0.164
Type of delivery (C-section)	1.777 (1.268–2.490)	**<0.001**
Preterm birth (no)	1.038 (0.654–1.645)	=0.875
Breastfeeding encouragement by medical staff when first holding the baby (yes)	1.595 (1.157–2.198)	**=0.004**
Rooming-in not practiced	2.320 (1.673–3.217)	**<0.001**
Told by community “you may not have good milk” (yes)	0.974 (0.669–1.416)	=0.889
Advised by community to give formula (yes)	0.673 (0.461–0.982)	**=0.040**
Pacifier from birth	5.027 (3.508–7.205)	**<0.001**
Pacifier after 6 weeks	4.767 (3.242–7.010)	**<0.001**

* The bold values are the statistically significant results.

## Data Availability

Data are contained within the article.
